# Lateral Approach and Plate Rod Sliding Humeral Osteotomy in Dogs—A Short Case Series

**DOI:** 10.3390/vetsci10020070

**Published:** 2023-01-18

**Authors:** William McCartney, Ciprian Ober, Maria Benito

**Affiliations:** 1 NOAH, 38 Warrenhouse Road, Baldoyle, Dublin D13 R2F6, Ireland; 2 Department of Surgery and Intensive Care, University of Agricultural Sciences and Veterinary Medicine, Calea Manastur 3-5, 400372 Cluj-Napoca, Romania; 3 Sandyford, Dublin D18, Ireland

**Keywords:** dog, dysplasia, elbow, humeral, osteotomy, sliding

## Abstract

**Simple Summary:**

Fragmented medial coronoid process is one of the most common diseases within the medial elbow joint compartment in dogs. Sliding humeral osteotomy was designed to translate the proximal humerus laterally, shifting the mechanical axis between the shoulder and carpus toward the lateral compartment of the elbow joint. A newly proposed technique using a lateral approach and plate/rod sliding humeral osteotomy was performed in five dogs. Lameness scores were improved in all dogs. This study might contribute to treatment strategies for medial compartment disease in dogs by offering an easier approach.

**Abstract:**

Five dogs of different breeds and ages were diagnosed with medial compartment disease of the elbow (MCDE). To resolve the condition, a modified technique using a lateral approach and plate/rod sliding humeral osteotomy (SHO) was considered. All dogs recovered uneventfully after surgery. There were no major complications, and all dogs were significantly improved compared to pre-operative condition. This novel technique of adding a pin, based on the alteration of the original technique, optimized resistance to fixation failure. An additional benefit was that the lateral approach was surgically familiar and easily allowed bone grafting. All five dogs treated with the novel approach had improved scores for pain and lameness. This study showed that SHO was more stable and less technically demanding with the addition of an intramedullary pin. This is the first report of a lateral approach and plate rod sliding humeral osteotomy to treat MCDE in dogs.

## 1. Introduction

Medial compartment disease of the elbow (MCDE) is an important cause of thoracic limb lameness in medium to large breed dogs [1,2,3], that includes elbow pathologies such as medial coronoid disease. In MCDE, there is damage or loss of articular cartilage, which can result in bone-to-bone contact. In some dogs with advanced disease, it is common that the cartilage is severely damaged or absent on the medial portion of the joint but appears relatively healthy on the lateral aspect [4].

Severe cartilage erosions occur with MCDE, and lateral joint compartment joint surfaces are normal. These aspects are similar to medial knee gonarthrosis in humans [5].

Thus, this type of elbow pathology can be treated by sliding humeral osteotomy (SHO), which facilitates the transfer of weight to the healthier lateral aspect of the joint [5]. The technique requires a medial approach of the humerus, and it was developed to unload the medial compartment of the canine elbow to improve lameness and pain. The technique also increases force plate measurement of limb loading in dogs with MCDE at 12–26 weeks after surgery [6,7]. Significant improvement in lameness in 60 elbows (46 dogs) by 12 weeks after SHO was also observed using force plate analysis [7]. The original SHO technique [1] was refined by Fitzpatrick et al. [7], and the surgery now uses custom-made plates and screws manufactured by New Generation Devices (Glen Rock, NJ, USA).

In relation to fixation, the plate/rod combination in dogs is an appropriate method to repair long bone fractures [6,8,9]. This combination was superior in reducing plate stress in comparison with the plate alone [6], and it can be successfully used to fix simple to complex diaphyseal fractures in dogs [8].

SHO case selection guidelines are still subjective, and the technique is demanding, especially for surgeons not very familiar with the approach and implant fixation [10]. The surgical approach to the medial humerus is also technically demanding because of important neurovascular structures on the bone [11]. The medial approach also involves cutting the pectoral muscle origins, retracting the biceps brachii muscle caudally, and the brachiocephalicus muscle cranially [12].

In this case series, we present a modified technique using a lateral approach and plate/rod sliding humeral osteotomy. We believe that this technique might be less technically demanding compared with the previous one.

## 2. Materials and Methods

Five dogs were referred to North Orthopedic Animal Hospital Dublin, Ireland, for diagnosis and treatment between 2019–2021. All necessary institutional guidelines for the use of animals were followed. Informed consent was obtained from the owners of the dogs. The animals included in this preliminary study were Labrador Retriever (2), Rottweiler (1), Rottweiler cross (1), and Golden Retriever (1). The weight range was 32–44 kg, and there were 3 males and 2 females. The age at surgery ranged from 3 to 6 years.

**Figure 1:**
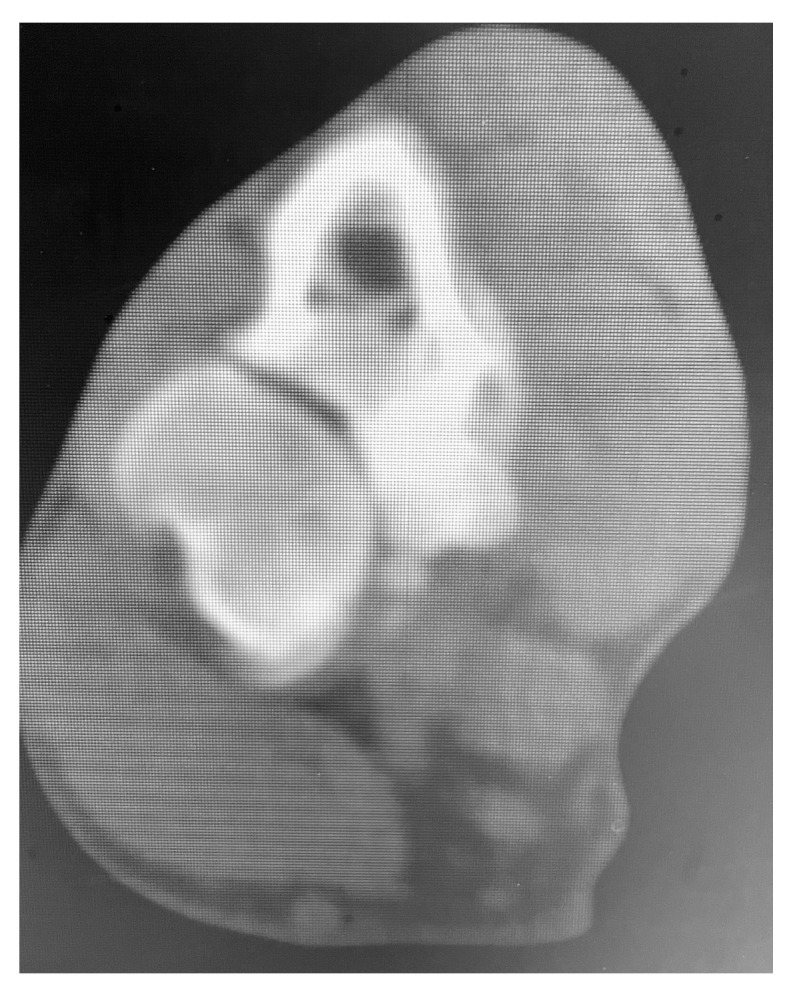
CT image of case 1 treated with MCDE. Note the small and misshapen medial coronoid process with decreased density and separated from the adjacent bone.

The dogs were selected for SHO based on poor or no response to conservative treatment. Lameness score, history, and preoperative imaging findings were recorded. All dogs presented varying degrees of lameness (three dogs’ grade 2 lameness and two dogs’ grade 3 lameness). Exacerbation of clinical signs with exercise was reported by all owners. All dogs presented the elbow slightly abducted and the antebrachium and manus in slightly external rotation. Orthopedic examination elicited a pain response on manipulation and palpation of the elbow joint in all dogs. Pain was obvious during deep palpation of the medial compartment of the joint, close to the tendon of insertion of the biceps brachii muscle. Flexion of the joint and supination of the distal limb induced a pain response also in all dogs. Joint effusion was palpated in three dogs (cases 1, 3, and 5). Enlargement of the joint, decreased range of motion, and crepitation was palpated in dogs 2 and 4 as the disease was more chronic. History and positive results of clinical and orthopedic examination highly raised suspicion for medial compartment elbow disease, and a CT scan (Siemens Emotion 1.25 mm slice) was performed for all dogs (Figure 1).

**Figure 2:**
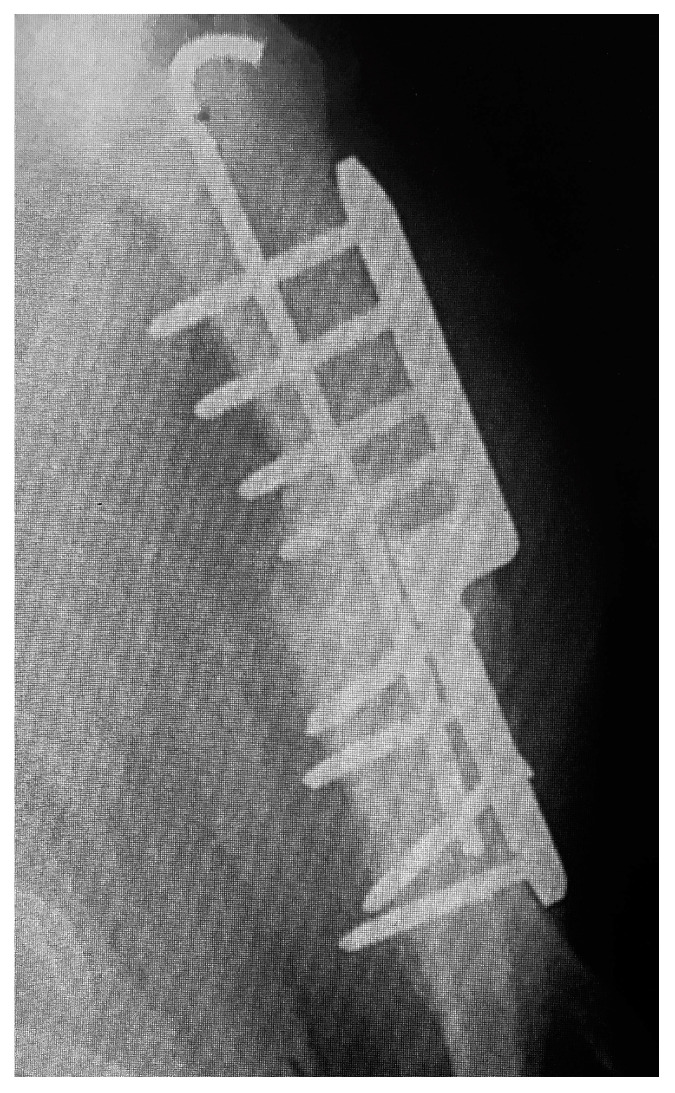
Post-operative view of the repair.

**Figure 3:**
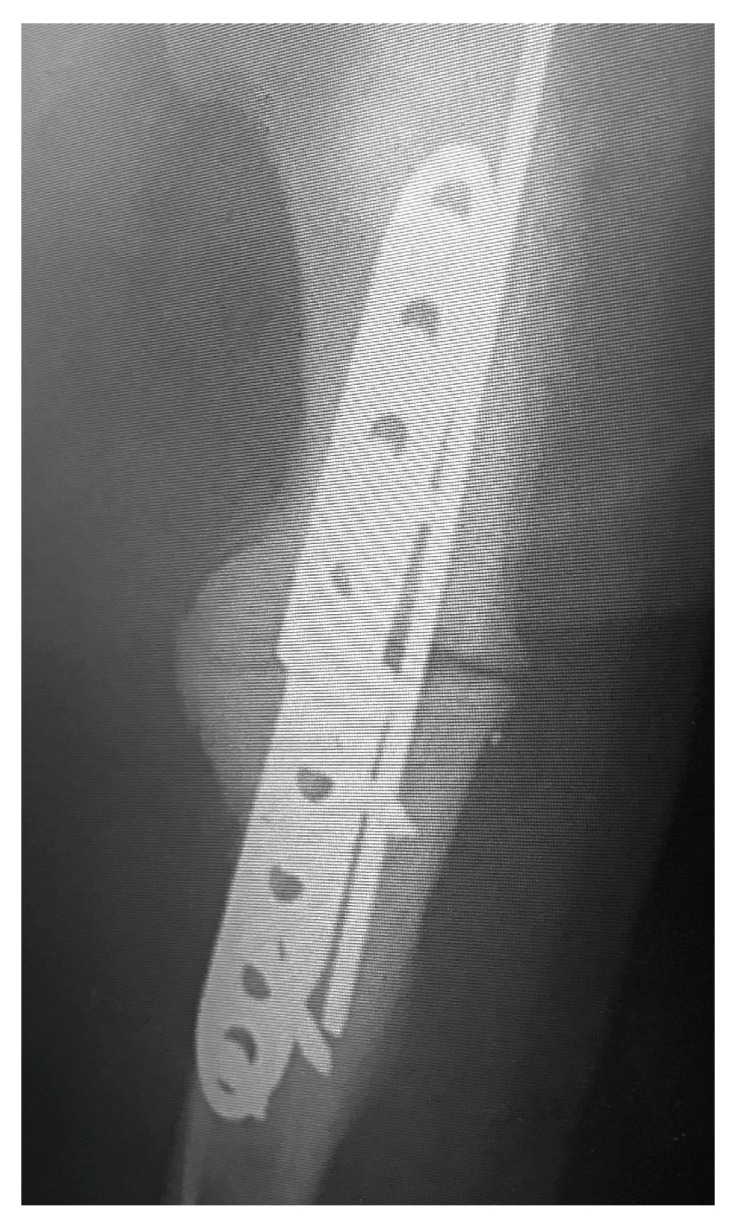
Post-operative view of callus formation.

Clinical examinations were also performed at 2, 6, 8, 12, and 14 weeks after surgery. Radiographs of the elbows were taken pre- and post-operative at 6 and 12 weeks (Figure 2 and Figure 3). The owners were asked to assess the dog using a telephone questionnaire (Canine Brief Pain Inventory).

The grading of complications was completed by the veterinary surgeon as either major (requiring surgery) or minor (requiring conservative treatment). A grading scale from 0 to 6 was used based on a telephone questionnaire with clients before and after surgery at 4, 6, 12, 16, 24, and 32 weeks. Outcome grading was classed as the final client response. The grading of the outcome was assessed as Grade 0: normal, no lameness and stiffness: Grade 1: once a week stiffness or lameness; Grade 2: intermittent (every 2–3 days) stiffness/lameness; Grade 3: frequent, once or twice a day stiffness; Grade 4: frequent 3–5 times daily lameness and stiffness; Grade 5: poor-everyday lame; Grade 6: fail (fixation failure). Before surgery, dogs were pre-medicated with medetomidine hydrochloride (10 mg/kg) (Domitor, Vetoquinol, Towcester, UK) and methadone hydrochloride (0.5 mg/kg) (Methadone, Alcami Corporation, Charleston, SC, USA). General anesthesia was induced with propofol (2 mg/kg) (Propofol-Lipuro, B. Braun Melsungen, Melsungen, Germany) and maintained with isoflurane (Isofane, Piramal Healthcare, Morpeth, UK). Intravenous amoxicillin/clavulanic acid (8.75 mg/kg) (Augmentin, GlaxoSmithKline, London, UK) was administered at induction. Evaluation of intraoperative analgesia was performed using heart rate, respiratory rate, noninvasive systolic blood pressure, hemoglobin saturation, carbon dioxide at the end of expiration, and concentration of inspired isoflurane [13].

Using the same SHO plate as previous publications, the humerus was approached from the craniolateral aspect in a standard approach. The plate was aligned with the humerus so that the distal aspect of the plate was proximal to the curvature of the distal humerus and the osteotomy line marked. After osteotomy, the intramedullary pin of 1.6 mm–2.4 mm was inserted retrograde into and exiting the proximal humerus. The intramedullary pin was inserted into the distal humerus. The aim was to have a pin approximately 30% of the humerus diameter. The SHO plate (New Generation Devices, Naples, FL, USA) was attached to the lateral aspect bone upside down to its normal position with the eight 3.5 mm locking screws, but the screws were left simply engaging the far cortex and not the plate., then the plate was clamped to the bone and the screws inserted fully to lock into the plate. Angling of some screws was at times necessary to avoid the pin. Postoperative analgesia was provided by meloxicam (0.2 mg/kg) (Metacam, Vetmedica GmbH, Boehringer Ingelheim, Ingelheim am Rhein, Germany) and methadone hydrochloride (0.5 mg/kg) (Methadone, Alcami Corporation, Charleston, SC, USA) for 48 h. The limb was bandaged using a Robert Jones bandage for 2–3 days after surgery. Dogs were allowed to do leash-only exercise 2–3 times daily for 12 weeks. Following 12 weeks, the owners were advised to gradually increase the level of free exercise over 6 weeks.

The first analysis summarized the data collected. An additional analysis examined the association between the pre and post-op grades. Due to the ordinal nature of the grading system, Spearman’s ranks correlation was used for the analysis. The final analysis compared the pre and post-operative grades to examine if there had been any changes between time points. Due to the ordinal nature of the scores, the analysis was performed using the Wilcoxon matched-pairs test. Continuous variables were summarized by mean, standard deviation, and data range. Categorical variables were summarized by the number and percentage in each category.

## 3. Results

A summary of the collected data is shown in Table 1. The association between the pre and post-op grades was examined. The correlation analysis gave a correlation coefficient of −0.41. However, this association was not statistically significant (*p* = 0.50), suggesting no strong evidence that the post-op scores were associated with the pre-op scores. It is noted that this analysis does not seem very appropriate, as the association between pre and post-op scores seems to be of limited interest. Further analysis compared the difference in pre and post-op grades. The statistical test found a statistically significant difference in grades between the two time points. The grades reduced over time, with all dogs being graded either 4 or 5 at the pre-operative time point and all dogs graded either 2 or 3 at the post-operative time point.

No major complication was recorded in any of the five dogs, and all recovered uneventfully after surgery. Minor complications included pin top seroma, wound breakdown, and minor infection. Intermittent stiffness and occasional lameness were noted after a discussion with the referring veterinary surgeon and the owners. Significant improvement was reported by the owners in all cases.

## 4. Discussion

In this case series, a new technique that allows the SHO procedure to be used by more surgeons by adding an intramedullary pin and using the more familiar craniolateral approach to the humerus was presented.

A previous study showed complication rates as high as 34% and major complications requiring revision surgery after SHO to treat medial compartment disease [1]. The technical difficulty and high complication rate might be responsible for the low number of cases treated with SHO [7]. An ex vivo study design [14] demonstrated that SHO was associated with significant alterations in thoracic limb alignment involving the elbow, mechanical axis of the humerus, radius/ulna, and mechanical axis of the thoracic limb, both in standing and recumbent limb positions. Another study [14] showed that medial compartment disease develops despite unloading of the medial compartment using a distal diaphyseal SHO in juvenile dogs with OC. Despite all these results, SHO ameliorates lameness and pain associated with MCDE [1,7]. Low complication rates and good clinical outcomes in the medium term after SHO were reported by a group from which a single surgeon performed all the procedures after a learning curve [7]. The key steps in SHO described by Fitzpatrick et al. [7] are plate positioning, screw placement parallel to the transcondylar axis, screw placement order, osteotomy exactly at the step, using the step as a guide and locking of all screws except those in holes 6 and 7. The order that the screws are locked was deemed to be necessary in a certain order. This was not necessary in the technique used in this study. There was no order of screw insertion in this study. Our results are comparable with the results reported in a previous study [15]. Most owners in that study reported diminished lameness at the last follow-up compared with preoperative status. The authors of the study observed no progression of osteoarthritis on the operated limb except in 1 dog out of 32. We also did not observe the progression of osteoarthritis at the final evaluation of the dogs in this case series. In a study evaluating dogs treated with the SHO procedure, the progression of the degenerative changes after the SHO technique in a small group of dogs was reported [15]. The assessment of the osteoarthritis progression in the study was done at 18–28 months after surgery, compared with our last follow-up at 3 months, and this might explain why no progression of degenerative changes was observed in our case series.

SHO is considered technically demanding, and it has been associated with a high incidence of complications in older studies also [16]. These complications have been significantly reduced to 4% minor complications using different implants and modifying techniques, as well as with the experience of the surgeon [7]. The problems associated with the technique are derived, maybe in part, from having to use the medial approach to the humerus and the fixation application. The original SHO technique consisted of only a medial plate and suffered a high incidence of failure. In this study, we have used an alteration of the SHO technique described by Fitzpatrick et al. (2015) [7]. The plate/rod fixation concept was introduced in this study to simplify the technique and decrease the rate of potential complications. A previous study [8] showed that the addition of a rod reduces plate strain and therefore resists loading more effectively and decreases the chance of implant failure. We believe the lateral approach is surgically familiar to most surgeons, and adding a pin also may decrease the chances of screw loosening and fixation failure. This modified SHO method of plate and rod fixation produced similar positive outcomes in these five cases but was less technically demanding. Cadaver studies in two dogs euthanatized for other reasons were carried out to be certain the proposed technique was feasible. This resulted in some refinement of technique, in particular, the retrograde insertion of the pin and the delay before the final full screw insertion.

The intramedullary plate-rod system is primarily indicated for complex fractures [17,18,19,20]. It has been shown that the intramedullary pin reduces the forces acting on the bone plate, which prevents screw loosening by increasing the fatigue life of the plate and facilitates the alignment of the bone shaft with the pin ideally being 30–40% of the bone diameter [6,19,21]. Bone plates have been used to bridge a simulated fracture gap in canine femurs, and the combination of a bone plate and an intramedullary pin have proved to be superior in reducing plate stress compared with the plate alone [6]. It was shown that a plate-rod combination represented a biomechanically superior fixation technique, compared with an interlocking nail, for the repair of comminuted tibial diaphyseal fractures [18]. The addition of an intramedullary pin reduces the stress applied to the plate, and as much as a 10-fold extension of the fatigue life of the bone plate can be achieved [6]. The pin can also assist in spatial aliniation of the limb and main fracture bones [9]. Sometimes unicortical placement of many of the plate screws is necessary because of the bone stock available [18]. Thus, plate-rod constructs can successfully be used for the repair of diaphyseal fractures of a wide range of severity in dogs and cats [8]. In our cases, the bone stock was available for the bicortical placement of all screws.

One limitation of the study is the small number of cases. Prospective studies using a larger population of dogs and longer-term follow-ups are necessary to assess the clinical outcome for SHO, as the new technique presented in this case series. We did not assess the outcome objectively, such as using force plate analysis, because of a lack of equipment, and this is another limitation of our study. The population included in these case series is represented by young and middle-aged dogs, and therefore we cannot have any conclusions about the potential benefits of the technique in older dogs. Nevertheless, we believe that these preliminary results are positive enough to warrant consideration. It is hoped that further prospective, controlled studies of this approach will add to improved knowledge and understanding of the MCDE treatment in dogs.

## 5. Conclusions

In this study, all five dogs affected with MCDE treated with the novel approach to SHO had improved scores for pain and lameness. This is the first report of a lateral approach and plate rod sliding humeral osteotomy to treat MCDE in dogs. We feel that this less demanding new approach and technique can be a good option for orthopedic surgeons in order to decrease pain and lameness secondary to end-stage medial compartment disease in dogs.

## Figures and Tables

**Table 1:** Summary of study data.

Factor	Category	Summary
Breed	Golden retriever	1 (20%)
	Labrador	2 (40%)
	Rottweiler	1 (20%)
	Rottweiler cross	1 (20%)
Sex	Female	2 (40%)
	Male	3 (60%)
Age	-	4.2 ± 1.3 [3, 6]
Weight (kg)	-	38.0 ± 4.7 [32, 44]
Pre-op grade	Grade 2	0 (0%)
	Grade 3	0 (0%)
	Grade 4	3 (60%)
	Grade 5	2 (40%)
Post-op grade	Grade 2	4 (80%)
	Grade 3	1 (20%)
	Grade 4	0 (0%)
	Grade 5	0 (0%)

Figures are: number (percentage) or mean ± standard deviation [data range]. Grade 0: normal no lameness and stiffness: Grade 1: once a week stiffness or lameness; Grade 2: intermittent (every 2–3 days) stiffness/lameness; Grade 3: frequent once or twice a day stiffness; Grade 4: frequent 3–5 times daily lameness and stiffness; Grade 5: poor-everyday lame; Grade 6: fail (fixation failure).

## Data Availability

Not applicable.
